# 550 Povidone Iodine Induced Apoptosis and Its Effect on Wound Healing in Human Dermal Fibroblasts

**DOI:** 10.1093/jbcr/iraf019.179

**Published:** 2025-04-01

**Authors:** Sam Jacob, Amina El Ayadi, Joseph Wenke

**Affiliations:** Shriners Children’s - Galveston; University of Texas Medical Branch; University of Texas Medical Branch and Shriners Children’s Texas

## Abstract

**Introduction:**

Povidone Iodine (Pv-I) is a broad-spectrum microbicide with high efficiency against bacteria, viruses, fungi, and protozoans. Due to its low bacterial resistance, Pv-I is commonly used for burn-wound debridement and as an antiseptic for the skin, mucosa, and rinsing body cavities. The Iodine molecule, which is in dynamic equilibrium with its carrier, polyvinylpyrrolidone, is released and oxidizes the amino acid groups exerting detrimental effects to microbes. Higher concentrations of Pv-I (5%-10%) is recommended for disinfection in clinical settings. However, in vitro evidence suggests its antibacterial activity at 0.1% without any host cell toxicity. Cytotoxicity of Pv-I in vivo and in vitro may be due to usage of higher than tolerable dose and subsequent presence of residual active iodine molecules. Paucity also exists on the adverse effects of Pv-I based on cell types and injury status of host cells. We hypothesize that higher dose of Pv-I will affect wound healing. Because fibroblasts are the principal cell type of the dermis, its vital role in wound healing, and the use of Pv-I in burn-wound debridement, the objective of this study is to examine the effect of Pv-I in wound healing in dermal fibroblasts using a conventional scratch assay.

**Methods:**

Confluent monolayer (passage 3-5) of primary normal human dermal fibroblasts, obtained commercially, were exposed at increasing concentrations of Pv-I ranging from 0.1% to 3.2% for 1 minute in culture dishes in a wound model of scratch assay and harvested 24 h after treatment. Cell viability was determined by MTT assay and apoptosis by flow cytometry using Annexin V. Effect of Pv-I on wound closure was calculated by computer-assisted quantitation of wound area and expressed as the remaining percentage of initial wound compared to controls at 24 h after treatment. Caspase-3 expression was examined using RT-PCR. Values are expressed as mean ±SD from 3 separate experiments (n=6).

**Results:**

No significant effect was noted in 0.1% Pv-I treated cells in all the parameters tested. Cell viability was significantly reduced by 32% and 90% at Pv-I concentrations of 0.2% and ≥ 0.8%, respectively (p< 0.05) compared to control. Cells started detaching at the 0.4% Pv-I dose. Flow cytometric analysis showed a significant increase in Annexin V positive cells with 0.2% Pv-I (114%, p=0.044) compared to control. The scratch assay indicated a significant decrease (2-fold, p=0.02) in wound closure in cells treated with 0.2% Pv-I compared to control. There was a 2-fold upregulation (p=0.04) in Caspase-3 expression at 0.2% Pv-I compared to control.

**Conclusions:**

Observed lower viability and increased apoptosis in caspase-3 pathway explains a mechanism for the reduced wound closure at 0.2% Pv-I in dermal fibroblasts, warrants further investigation.

**Applicability of Research to Practice:**

Because dermal fibroblasts play a vital role in wound healing, the adverse effects of Pv-I need more studies to establish an optimum dose to balance biocidal activity.

**Funding for the Study:**

N/A

